# Risk factors associated with the development of multidrug-resistant bloodstream infection in patients with colonization: a case-control retrospective study

**DOI:** 10.1016/j.ijregi.2026.101016

**Published:** 2026-09-15

**Authors:** Jinan Al Jadeedi, Majada Allouyahi, Rouya Al Busaidi, Shatha Al Riyami, Abdullah Balkhair

**Affiliations:** 1 College of Medicine and Health Sciences, Sultan Qaboos University, Muscat, Oman; 2 Infectious Diseases Unit, Department of Medicine, Sultan Qaboos University Hospital, University Medical City, Muscat, Oman

**Keywords:** Colonization, Bloodstream infection, Multidrug-resistant organisms, Oman

## Abstract

**Objectives:**

To examine the risk factors associated with the development of multidrug-resistant organism (MDRO) bloodstream infection (BSI) in hospitalized adult patients with previous MDRO colonization or isolation from clinical samples.

**Methods:**

This retrospective, matched case-control study compared patients with previous MDRO colonization or isolation from clinical samples who developed BSI with patients with the same MDRO species who did not develop BSI. Factors associated with the progression from previous MDRO isolation to BSI were examined using conditional logistic regression, stratified by the matched set. Matching was performed at 1:2 according to age and sex.

**Results:**

A total of 1269 non-repeat previous MDRO isolation events were identified in 961 patients. A total of 12.9% of patients had two or more MDROs isolates. Overall, 172 (13.5%) non-repeat BSI events occurred in patients with MDRO colonization/infection, of which 78 (45.4%) were caused by the same MDRO species. A total of 6.1% of previous MDRO isolation events progressed to BSI caused by the same MDRO species. Previous admission to the intensive care unit (adjusted odds ratio 4.31; 95% confidence interval 1.44-12.96; *P* = 0.009) and presence of a central venous catheter (adjusted odds ratio 3.38; 95% confidence interval 1.55-7.39; *P* = 0.002) were independent risk factors associated with the development of BSI caused by the same MDRO species.

**Conclusion:**

Recent intensive care unit admission and central venous catheterization are independently associated with progression from previous MDRO isolation to BSI.

## Highlights

• Colonization with multidrug-resistant organism (MDRO) is an important risk factor for the subsequent development of bloodstream infection (BSI).
• A total of 12.9% of patients were co-colonized with two or more MDROs.
• A total of 6.1% of MDRO colonization events progressed to BSI by the same colonizing MDRO.
• Concordance between MDRO colonization and subsequent BSI was 45.3% of BSI events.
• Progression from MDRO carriage to BSI independently associated with intensive care unit and central venous catheter.

## Introduction

Multidrug-resistant organisms (MDROs) such as carbapenem-resistant *Klebsiella pneumoniae* (CRKP), carbapenem-resistant *Acinetobacter baumannii* (CRAB), methicillin-resistant Staphylococcus aureus (MRSA), and vancomycin-resistant *Enterococcus faecium* (VRE) are considered emerging threats worldwide, representing an ongoing global health burden [1]. CRKP and CRAB are classified as critical priority pathogens, whereas MRSA and VRE are classified as high-priority pathogens on the World Health Organization Bacteria Priority Pathogen List of 2024 [2].

A study on the epidemiology of MDROs in a teaching hospital in Oman found an increasing trend in the prevalence rates of MDRO during the study period, with a rate of 11.2 per 1000 admissions [3]. A more recent study on the prevalence of MDRO in Oman showed a growing trend during the study period between 2017 and 2022, with extended-spectrum β-lactamase isolates being the primary MDRO pathogens, followed by CRAB, MRSA, CRKP, and VRE [4].

Screening for MDRO colonization using active surveillance cultures is a common practice substantiated by extensive evidence, especially in high-risk settings [5]. Colonization is often a prerequisite for infection [6], but the risk of infection after MDRO colonization remains unknown.

Studies have shown that colonization with MDROs significantly increases the risk of subsequent infection, with risk ratios varying by organism; however, the timing and factors triggering this transition are not fully understood [7,8]. A systematic review found that 7.6%-44.4% of patients colonized with carbapenem-resistant *Enterobacterales* developed clinical infections [9]. Another systematic review and meta-analysis reported an incidence rate of 23.2% for CRKP infection after colonization [10]. Similarly, colonization with MRSA significantly increases the risk of subsequent MRSA infection, with 7.0% of patients who acquired MRSA during their inpatient stay developing an infection [11]. Likewise, colonization with VRE increases the risk of VRE-related bacteremia, with 4.76% of VRE-colonized patients developing bacteremia in one study [12]. Lastly, a systematic review and meta-analysis of nine studies examining CRAB colonization and risk factors for subsequent infection showed that invasive CRAB infection was significantly higher among patients colonized with CRAB, with an odds ratio (OR) of 11.14 compared with non-carriers [13].

A study from Saudi Arabia found that colonization was associated with an increased risk of infection with CRKP, MRSA, and VRE, with risk ratios of 4.6, 8.2, and 22, respectively [14]. Another study from Greece on patients with hematologic malignancies undergoing transplantation or cellular therapy showed that colonization with carbapenem-resistant gram-negative bacilli and VRE was associated with concordant bloodstream infections (BSIs) in 18.7% and 4.4% of patients, respectively [15].

Predicting the infection risk in patients colonized with MDRO may help improve timely and appropriate antibiotic therapy [16]. Clinical factors for subsequent MDRO infection in patients colonized with MDROs include diabetes mellitus, previous invasive procedures, and antibiotic exposure [17]. Other risk factors for progression from MDRO colonization to invasive infection include admission to the intensive care unit (ICU), invasive procedures, and multisite colonization [10].

Although it has been established that previous MDRO colonization correlates with an increased risk of subsequent infection, the extent to which this association is observed in our patient population remains largely unknown. Understanding these risk factors is crucial for guiding clinicians in the prompt initiation of empiric MDRO pathogen coverage in adult patients with MDRO colonization with sepsis or septic shock and a high risk of BSI by the same MDRO species [18].

We hope that this research will help us answer the following question: Does previous colonization with CRKP, CRAB, MRSA, and VRE increase the risk of BSI with these isolates in our clinical and epidemiologic settings?

## Methods

### Study design and population

We performed a single-center, retrospective, observational, matched case-control study of adult (aged ≥18 years) patients with a positive surveillance screen for CRKP, CRAB, MRSA, or VRE from January 1, 2022 to June 30, 2024 to identify risk factors associated with development of BSI caused by the same MDRO species. The cases consisted of patients with previous MDRO isolation who subsequently developed BSI by the same MDRO isolate within 365 days of the first known MDRO isolation, with MDRO isolation documented at least 48 hours before blood culture collection. The control group was composed of patients with previous MDRO isolation who did not develop BSI by the same MDRO isolate within 365 days of the first known previous MDRO isolation. Matching was performed at a ratio of 1:2 according to age (±2 years) and sex, and computer-generated random sampling was used when more than two eligible controls were available.

### Setting

This study was conducted at Sultan Qaboos University Hospital, a large tertiary care academic hospital in Oman. Active surveillance cultures (nasal swabs for MRSA screening and rectal swabs for CRKP, CRAB, and VRE) were routinely performed for hospitalized patients with at least one of the following criteria: (i) admission to a high-risk unit, including the ICU, high-dependency unit, and hematology ward; (ii) patients transferred from other hospitals or long-term care facilities; and (iii) patients admitted for major orthopedic or cardiac surgery.

### Data

Data were collected retrospectively using standardized electronic case report forms. Comorbidities, including diabetes mellitus, chronic kidney disease, chronic liver disease, chronic lung disease, cardiovascular disease, malignancy, neutropenia, immunosuppression, solid organ transplantation, and hematopoietic stem cell transplantation, were also recorded. Health care exposures, including previous hospitalization within 90 days; previous ICU admission within 90 days; hemodialysis; and surgery and procedures, including central venous catheterization, urinary catheterization, endotracheal intubation, and nasogastric or gastrostomy tube placement, were documented. The risk factor variables were considered from the time of the first known MDRO isolation to the onset of a BSI (cases) or the lapse of 365 days from the first known MDRO isolation without a BSI (controls).

### Definitions

MDRO colonization was defined as the isolation of CRKP, CRAB, MRSA, or VRE from active surveillance screens. Previous MDRO isolation was defined as recovery of an MDRO from any surveillance or clinical specimen, excluding blood cultures, during the specified period preceding the index BSI. In addition, clinical samples (other than blood cultures) with positive cultures for CRKP, CRAB, MRSA, or VRE before a BSI event were also included. For the purpose of this study, MDRO colonization refers to either a positive surveillance culture or previous MDRO isolation from clinical samples excluding blood cultures. MDRO BSI was defined by blood culture positivity with the same MDRO isolate, occurring within 365 days of first documented MDRO isolation, with the isolate documented at least 48 hours before blood culture collection. The onset of MDRO BSI was considered to be the date of index blood culture collection.

### Statistical analysis

Categorical variables are expressed as frequencies and percentages and continuous variables as mean ± SD for normally distributed data or as median with interquartile range (IQR) for non-normally distributed data. Time-restricted analysis assessing temporal proximity of subsequent BSI events occurring within ≤90 and ≤180 days after documented MDRO isolation was performed. Associations between potential risk factors and MDRO BSIs were assessed using univariable conditional logistic regression. Variables associated with MDRO BSI in the univariable analysis (*P* < 0.10) were included in the multivariable conditional logistic regression model. An exploratory analysis of cumulative risk factor burden was performed, and the associations with BSI was assessed using conditional logistic regression. The number of risk factors was modeled as a continuous variable. ORs with 95% confidence intervals (CIs) were reported, and *P* < 0.05 was considered statistically significant. Statistical analyses were performed using jamovi (Version 2.6.44; The jamovi project, 2025).

## Results

A total of 961 patients had previous MDRO isolation with at least one multidrug-resistant organism. Among them, 124 (12.9%) patients were co-colonized or co-infected with two or more MDROs. A total of 1269 non-repeat previous MDRO isolation events were recorded. VRE was the most common MDRO isolate from surveillance screens, accounting for 463 (36.5%) episodes. This was followed by CRKP (390 events, 30.7%), MRSA (260 events, 20.5%), and CRAB (156 events, 12.3%). A total of 157 (16.3%) patients developed at least one MDRO BSI, of whom 15 (9.6%) had two or more non-repeat MDRO BSIs, resulting in 172 BSI events (Table 1).

**Table 1:** Distribution of MDRO colonization/isolation and BSI.

Characteristic	n (%)
MDRO colonization	
Patients colonized with ≥1 MDRO	961
Patients co-colonized with ≥2 MDROs	124 (12.9%)
Total MDRO colonization events (non-repeat)	1269
Vancomycin-resistant *enterococci* (VRE)	463 (36.5%)
Carbapenem-resistant *Klebsiella pneumoniae* (CRKP)	390 (30.7%)
Methicillin-resistant *Staphylococcus aureus* (MRSA)	260 (20.5%)
Carbapenem-resistant *Acinetobacter baumannii* (CRAB)	156 (12.3%)
MDRO bloodstream infection	
Patients with MDRO bloodstream infection	157
Patients with ≥2 MDRO bloodstream infections (non-repeat)	15 (9.6%)
MDRO bloodstream infection events (non-repeat)	172
Carbapenem-resistant *Klebsiella pneumoniae* (CRKP)	73 (42.4%)
Methicillin-resistant *Staphylococcus aureus* (MRSA)	45 (26.2%)
Vancomycin-resistant *enterococci* (VRE)	33 (19.2%
Carbapenem-resistant *Acinetobacter baumannii* (CRAB)	21 (12.2%)
MDRO bloodstream infection events caused by the same colonizing MDRO	78 (45.3%)
Carbapenem-resistant *Klebsiella pneumoniae* (CRKP)	40 (51.3%)
Vancomycin-resistant *enterococci* (VRE)	14 (17.9%)
Methicillin-resistant *Staphylococcus aureus* (MRSA)	14 (17.9%)
Carbapenem-resistant *Acinetobacter baumannii* (CRAB)	10 (12.8%)
Concordance between MDRO colonization and subsequent bloodstream infection	n/N (%)
Carbapenem-resistant *Klebsiella pneumoniae* (CRKP)	40/390 (10.2%)
Carbapenem-resistant *Acinetobacter baumannii* (CRAB)	10/156 (6.4%)
Methicillin-resistant *Staphylococcus aureus* (MRSA)	14/260 (5.4%)
Vancomycin-resistant *enterococci* (VRE)	14/463 (3.0%)

Abbreviations: BSI, bloodstream infection; MDRO, multidrug-resistant organism.

Among these MDRO BSIs, CRKP accounted for 73 (42.4%), MRSA for 45 (26.2%), VRE for 33 (19.2%), and CRAB for 21 (12.2%) cases.

Of the 172 MDRO BSI events, 78 (45.4%) were caused by the same MDRO isolate. Specifically, 40 (51.3%) of these infections were due to CRKP, 14 (17.9%) were due to VRE, 14 (17.9%) were due to MRSA, and 10 (12.8%) were due to CRAB. The concordance rates between previous MDRO isolation and subsequent BSI were as follows: CRKP showed the highest concordance, with 40 infections of 390 events (10.2%), followed by CRAB with 10 of 156 (6.4%), MRSA with 14 of 260 (5.4%), and VRE with 14 of 463 (3.0%).

Among the 124 cases of MDRO co-isolation, the most frequent combination was CRKP and VRE, observed in 45 (36.3%) patients. This was followed by a combination of MRSA, VRE, CRAB, and CRKP, which accounted for 16 cases (12.9%). Co-isolation of CRAB, CRKP, and VRE occurred in 15 (12.1%) patients. The combination of CRKP and MRSA was found in 11 (8.9%) cases, which was the same number as that for CRAB and VRE co-isolation. CRAB and MRSA co-isolation was identified in five (4.0%) patients. Less common combinations included CRAB, MRSA, and VRE in three (2.4%) cases; CRKP, MRSA, and VRE in one (0.8%) case; and CRAB, CRKP, and MRSA in one (0.8%) Case.

Table 2 depicts demographic characteristics and clinical outcomes of patients with multidrug resistant bloodstream infection and matched controls. Among the cases (n = 78) and controls (n = 156), the proportion of males was similar, with 59% (46 of 78) in the cases and 56% (88 of 156) in the controls (*P* = 0.709), and the median age was identical in both groups at 64.5 years (IQR 43-73; *P* = 0.962).

**Table 2:** Demographic characteristics and clinical outcomes of patients with multidrug-resistant BSI and matched controls, stratified by organism.

Characteristic	Overall cases	Overall controls	*P-*value	CRKP cases	CRKP controls	VRE cases	VRE controls	MRSA cases	MRSA controls	CRAB cases	CRAB controls
Baseline characteristics											
MDRO, (%)	78 (100%)	156 (100%)	—	40 (51%)	80 (51%)	14 (18%)	28 (18%)	14 (18%)	28 (18%)	10 (13%)	20 (13%)
Male sex, n (%)	46 (59%)	88 (56%)	0.709	24 (60%)	42 (53%)	10 (71%)	20 (71%)	9 (64%)	20 (71%)	3 (30%)	6 (30%)
Age, median (IQR), years	64.5 (43-73)	64.5 (43-73)	0.962	70 (52-76)	69.5 (50-76)	60.5 (41-68)	60.5 (39-67)	55.5 (42-71)	55 (42-73)	64.5 (41-72)	64.5 (41-71)
Clinical outcomes											
Length of stay, median (IQR), days	36.5 (13-71)	18 (7-36.5)	<0.001	36.5 (16-69)	20 (7-37)	73 (60-116)	19 (7-57)	10 (3-21)	9.5 (3-23)	50 (28-91)	26.5 (18-32)
Time from colonization to BSI, median (IQR), days^a^	11 (1-97)	—	—	12.5 (1-116)	—	14.5 (4-47)	—	1.5 (1-217)	—	16.5 (3-140)	—
Proportion of subsequent BSI events occurring within ≤90 days, %	74.4%	—	—	72.5%	—	85.7%	—	71.4%	—	70%	—
Proportion of subsequent BSI events occurring within ≤180 days, %	80.8%	—	—	82.5%	—	85.7%	—	71.4%	—	80%	—
30-Day mortality, n (%)	27 (34.6%)	4 (2.6%)	<0.001	15 (38%)	1 (1%)	4 (29%)	0	5 (36%)	2 (7%)	3 (30%)	1 (5%)

Abbreviations: MDRO: multidrug-resistant organism; BSI, bloodstream infection; CRAB, carbapenem-resistant *Acinetobacter baumannii*; CRKP, carbapenem-resistant *Klebsiella pneumoniae*; MRSA, methicillin-resistant *Staphylococcus aureus*; VRE, vancomycin-resistant *Enterococcus faecium*; IQR: interquartile range.

a Applicable to cases only.

Among the 78 cases and 156 matched controls, the distribution of MDRO was as follows: CRKP, 51%; VRE, 18%; MRSA, 18%; and CRAB, 13%.

The proportion of males varied across the MDRO types. In these cases, males represented 71%, 64%, 60%, and 30% of VRE, MRSA, CRKP, and CRAB infections, respectively. The median age of the patients was 70 (5276) years for CRKP, 64.5 (IQR 41-72) years for CRAB, 60.5 (41-68) years for VRE, and 55.5 (42-71) years for MRSA.

The cases had a significantly longer median length of hospital stay than the controls (36.5 days [IQR 13-71] vs 18 days [IQR 7-36.5]) (*P* < 0.001). The median time from previous MDRO isolation to BSI was 11 (IQR 1-97) days. Of the 78 subsequent BSI episodes, 58 (74.4%) occurred within 90 days and 63 (80.8%) within 180 days of the first documented MDRO isolation.

The median time from previous MDRO isolation to BSI was 16.5 days (3-140) for CRAB, 12.5 (1-116) days for CRKP, 14.5 (4-47) days for VRE, and 1.5 (1-217) days for MRSA, with no significant difference (*P* = 0.721). At ≤90 days, the proportions were 70.0% (seven of 10) for CRAB, 72.5% (29 of 40) for CRKP, 71.4% (10 of 14) for MRSA, and 85.7% (12 of 14) for VRE. At ≤180 days, the corresponding proportions were 80.0% (eight of 10), 82.5% (33 of 40), 71.4% (10 of 14), and 85.7% (12 of 14), respectively.

The 30-day mortality rate was notably higher in the cases (34.6%, 27 of 78) than in the controls (2.6%, four of 156) (*P* < 0.001). Patients with BSIs secondary to CRKP had the highest 30-day mortality (38%), followed by those with MRSA (36%), whereas those with VRE BSIs had the lowest (29%), albeit with exceptionally high mortality. The 30-day mortality rate for patients with CRAB BSIs was 30%.

The association between the development of MDRO BSIs in patients with previous MDRO isolation and comorbidities, health care exposures, and procedures among cases and controls was analyzed (Table 3). Chronic kidney disease was significantly more common in the cases (42.3%) than in the controls (23.7%) (OR 2.54; 95% CI 1.36-4.77, *P* = 0.004). Cardiovascular disease was also more frequent in cases (52.6%) than in controls (35.3%) (OR 2.67; 95% CI 1.35-5.28, *P* = 0.005). Likewise, neutropenia occurred in 17.9% of cases vs 5.8% of controls (OR 4.00; 95% CI 1.51-10.56, *P* = 0.005). The prevalence of diabetes mellitus, chronic liver disease, chronic lung disease, malignancy, and immunosuppression did not differ significantly among the groups.

**Table 3:** Univariable conditional logistic regression analysis of factors associated with bloodstream infection in patients with previous MDRO isolation.

	Cases (n = 78)	Controls (n = 156)	OR (95% CI)	*P*-value
Comorbidities, n (%)				
Diabetes mellitus	31 (39.7%)	71 (45.5%)	0.77 (0.43-1.37)	0.374
Chronic kidney disease	33 (42.3%)	37 (23.7%)	2.54 (1.36-4.77)	**0.004**
Chronic liver disease	10 (12.8%)	12 (7.7%)	1.77 (0.72-4.31)	0.212
Chronic lung disease	20 (25.6%)	36 (23.1%)	1.17 (0.60-2.32)	0.642
Cardiovascular disease	41 (52.6%)	55 (35.3%)	2.67 (1.35-5.28)	**0.005**
Malignancy	14 (17.9%)	31 (19.9%)	0.86 (0.40-1.85)	0.703
Neutropenia	14 (17.9%)	9 (5.8%)	4.00 (1.51-10.56)	**0.005**
Immunosuppression	13 (16.7%)	19 (12.2%)	1.46 (0.67-3.18)	0.343
Health care exposures/procedures, n (%)				
Previous hospitalization within 90 days	42 (53.8%)	91 (58.3%)	0.83 (0.47-1.45)	0.505
Previous ICU admission within 90 days	15 (19.2%)	12 (7.7%)	3.18 (1.32-7.65)	**0.010**
Hemodialysis	23 (29.5%)	20 (12.8%)	2.74 (1.39-5.40)	**0.004**
Surgery	28 (35.9%)	34 (21.8%)	2.11 (1.12-3.97)	**0.021**
Presence of central venous catheter	46 (59.0%)	43 (27.6%)	4.00 (2.14-7.47)	**0.000**
Presence of a Foley catheter	34 (43.6%)	62 (39.7%)	1.20 (0.66-2.18)	0.541
Endotracheal intubation	38 (48.7%)	49 (31.4%)	2.21 (1.23-3.96)	**0.008**
Presence of nasogastric or gastrostomy tube	38 (48.7%)	65 (41.7%)	1.37 (0.77-2.45)	0.282

Abbreviations: ICU, intensive care unit; MDRO, multidrug-resistant organism; OR, odds ratio.

Boldface indicates statistically significant results

Regarding health care exposures and procedures, the presence of a central venous catheter (OR 4.00; 95% CI 2.14-7.47, *P* = 0.000), previous ICU admission within 90 days (OR 3.18; 95% CI 1.32-7.65, *P* = 0.010), hemodialysis (OR 2.74; 95% CI 1.39-5.40, *P* = 0.004), surgery within the previous 90 days (OR 2.11; 95% CI 1.12-3.97, *P* = 0.021), and endotracheal intubation (OR 2.21; 95% CI 1.23-3.96, *P* = 0.008) were all found to be significantly associated with MDRO BSIs. Other exposures, such as previous hospitalization within 90 days, did not differ significantly between the groups.

Multivariable conditional logistic regression analysis showed that previous admission to the ICU within 90 days (adjusted OR 4.31; 95% CI 1.44-12.96; *P* = 0.009) and presence of a central venous catheter (adjusted OR 3.38; 95% CI 1.55-7.39; *P* = 0.002) were independent risk factors associated with the development of MDRO BSIs caused by the same MDRO species (Table 4). A progressive increase in the odds of BSI was observed with increasing risk factor burden. Compared with none to one risk factor, two to three factors were associated with OR 4.39 (95% CI 1.95-9.89; *P* < 0.001), and four or more factors with OR 14.59 (95% CI 5.53-38.47; *P* < 0.001). Each additional risk factor was associated with OR 1.87 (95% CI 1.49-2.36; *P* < 0.001). In the focused ICU/central venous catheter analysis, the combination of both factors was associated with OR 14.67 (95% CI 3.5-60.38; *P* < 0.001).

**Table 4:** Multivariable conditional logistic regression analysis of factors independently associated with bloodstream infection in patients with previous MDRO isolation.

	Cases (n = 78)	Controls (n = 156)	Adjusted OR (95% CI)	*P*-value
Comorbidities, n (%)				
Chronic kidney disease	33 (42.3%)	37 (23.7%)	1.64 (0.75-3.62)	0.217
Cardiovascular disease	41 (52.6%)	55 (35.3%)	2.16 (0.95-4.92)	0.067
Neutropenia	14 (17.9%)	9 (5.8%)	1.99 (1.44-6.88)	0.277
Health care exposures/procedures, n (%)				
Previous ICU admission within 90 days	15 (19.2%)	12 (7.7%)	4.31 (1.44-12.96)	**0.009**
Hemodialysis	23 (29.5%)	20 (12.8%)	1.44 (0.60-3.49)	0.415
Surgery	28 (35.9%)	34 (21.8%)	2.12 (1.00-4.52)	0.051
Presence of central venous catheter	46 (59.0%)	43 (27.6%)	3.38 (1.55-7.39)	**0.002**
Endotracheal intubation	38 (48.7%)	49 (31.4%)	1.16 (0.57-2.38)	0.680

Abbreviations: CI, confidence interval; ICU, intensive care unit; OR, odds ratio.

Boldface indicates statistically significant results

A pathogen-specific subgroup analysis was performed for patients with previous isolation of CRKP, comprising 40 cases and 80 matched controls (Table 5). Among patients with previous isolation of CRKP, central venous catheterization was the strongest factor associated with subsequent BSI in the univariable analysis (OR 7.43, 95% CI 2.80-19.66; *P* < 0.001). Chronic kidney disease (OR 2.08, 95% CI 0.88-4.90; *P* = 0.095), hemodialysis (OR 2.23, 95% CI 0.96-5.18; *P* = 0.061), urinary catheterization (OR 2.11, 95% CI 0.92-4.81; *P* = 0.076), and endotracheal intubation (OR 2.12, 95% CI 0.99-4.56; *P* = 0.053) showed weaker associations. In the multivariable model, central venous catheterization remained independently associated with CRKP BSI (adjusted OR 7.72, 95% CI 2.44-24.50; *P* < 0.001), whereas the other variables were not statistically significant. Pathogen-specific analyses were not performed for MRSA, VRE, or CRAB because of the small number of matched case sets (14, 14, and 10 cases, respectively).

**Table 5:** Carbapenem-resistant *Klebsiella pneumoniae*–specific univariable and multivariable matched analyses (40 cases, 80 matched controls).

Variable	Unadjusted OR	95% CI	*P*-value	Adjusted OR	95% CI	*P*-value
Diabetes mellitus	0.77	0.36-1.67	0.512	—	—	—
Chronic kidney disease	2.08	0.88-4.90	0.095	1.00	0.32-3.09	0.997
Chronic liver disease	0.87	0.24-3.11	0.832	—	—	—
Chronic lung disease	0.54	0.19-1.53	0.248	—	—	—
Cardiovascular disease	1.15	0.47-2.86	0.758	—	—	—
Malignancy	0.60	0.19-1.89	0.382	—	—	—
Neutropenia	3.35	0.59-18.87	0.171	—	—	—
Immunosuppression	1.40	0.46-4.29	0.558	—	—	—
Previous hospitalization within 90 days	0.95	0.44-2.06	0.896	—	—	—
Previous ICU admission within 90 days	2.00	0.65-6.20	0.230	—	—	—
Hemodialysis	2.23	0.96-5.18	0.061	0.80	0.24–2.63	0.713
Surgery	1.93	0.85-4.40	0.118	—	—	—
Central venous catheter	**7.43**	**2.80-19.66**	**<0.001**	**7.72**	**2.44-24.50**	**<0.001**
Urinary catheter	2.11	0.92-4.81	0.076	1.85	0.67-5.11	0.234
Endotracheal tube	2.12	0.99-4.56	0.053	0.91	0.34-2.41	0.842
Nasogastric/gastrostomy tube	1.80	0.84-3.85	0.131	—	—	—

Note: The adjusted model included chronic kidney disease, hemodialysis, central venous catheter, urinary catheter, and endotracheal tube.

Abbreviations: CI, confidence interval; ICU, intensive care unit; OR, odds ratio.

Boldface indicates statistically significant results

## Discussion

Colonization with or previous isolation of MDRO is an important risk factor for the subsequent development of BSI by the same MDRO pathogens [19]. However, colonization alone is insufficient to cause infection. In this retrospective matched case-control study, we found that 10.2%, 6.4%, 5.4%, and 3.0% of patients with previous isolation of CRKP, CRAB, MRSA, and VRE, respectively, developed BSI due to the same MDRO species. The progression from previous MDRO isolation to BSI was independently associated with recent admission to a critical care unit and with central venous catheterization.

With the exception of CRAB, the observed rates of progression to BSI were broadly consistent with those reported in most previous studies [7,20]. The lower progression to CRAB BSI observed in our study may be partly explained by differences in cohort composition and screening methods. Although multisite and respiratory tract colonization and care in the ICU have been associated with an increased risk of subsequent CRAB BSI, our cohort included patients admitted to the ICU and those who were not, with surveillance limited to a single rectal screen. This limitation may have contributed to the lower observed risk of progression.

Although VRE was the most frequently observed MDRO isolate (36.5%) in the examined cohort, its subsequent progression to BSI was the least frequent (3.0%). In contrast, CRAB was the least observed colonizing MDRO (12.3%), but it exhibited a higher rate of progression to BSI (6.4%). Both findings are consistent with recent reports from Greek and Italian cohorts, citing BSI progression in patients with VRE and CRAB colonization of 3.5% and 4.2% and 19.7% and 20%, respectively [7,20]. These findings suggest that the epidemiologic burden of previous MDRO isolation does not necessarily parallel the risk of progression to BSI because this is determined by the virulence of the MDRO and the patient’s risk factors.

The overall rate of BSI in this study was 13.5% (172 BSI events in 1269 colonization/previous isolation events). Notably, concordance between surveillance MDRO and BSI cultures was observed in 45.3% of BSIs events, with CRKP and CRAB exhibiting the highest rates at 10.2% and 6.4%, respectively. This observation is consistent with the existing literature, indicating that a substantial fraction of MDRO BSIs is not attributed to the same colonizing strain [21]. This observation may be influenced by the limited sensitivity of the surveillance methods used, including those used in this study.

Interestingly, 54.7% of MDRO BSI events occurred in patients with negative screens for the culprit MDRO, reflecting the limitations of single-site, culture-based surveillance methods. Similarly, other studies have highlighted the limited sensitivity of this method, with only 23% of those who developed MDRO infection having a positive surveillance culture beforehand in one study [22], whereas 61% of patients who developed MDRO BSI had a matched MDRO screen in another study [23].

Co-isolation of multiple MDROs is increasingly recognized and may reflect shared risk factors. In our cohort, co-isolation with CRKP and VRE was the predominant co-isolation event, accounting for 36.3% of all MDRO co-isolation events. These findings suggest an association between CRKP and VRE, which share an ecological niche within the gastrointestinal tract, resulting from antimicrobial exposure and disruption of the intestinal microbiota [24].

The median interval from documented previous MDRO isolation to BSI was 11 days in our study, with approximately three-quarters of subsequent BSIs occurring within 90 days of MDRO isolation, suggesting that the first 3 months after colonization/MDRO isolation represents an important period of risk, signifying that progression to BSI frequently occurs relatively early after MDRO isolation. Similarly, Chu et al*.* [25] reported a median interval of 6 days from CRKP intestinal colonization to BSI, supporting the concept that patients with colonization are particularly at risk of progression to BSI during the early period after MDRO carriage.

The current study demonstrated that progression from previous MDRO isolation to BSI was associated with a significantly longer median hospital stay (36.5 vs 18 days). This finding is consistent with several studies, including the PANORAMA prospective cohort study [26]. Likewise, 30-day mortality was markedly higher among cases than among controls (34.6% vs 2.6%), emphasizing the serious consequences of progression from previous MDRO isolation to BSI. This mortality rate is consistent with that of a recent systematic review that reported an overall mortality rate of 32% [27].

Our study identified several risk factors associated with the progression from previous MDRO isolation to BSI. Chronic kidney disease, cardiovascular disease, neutropenia, hemodialysis, recent surgery, endotracheal intubation, previous ICU admission, and central venous catheterization were associated with subsequent MDRO BSI in univariable analyses; nevertheless, only recent ICU admission (adjusted OR 4.31; 95% CI 1.44-12.96; *P* = 0.009) and the presence of a central venous catheter (adjusted OR 3.38; 95% CI 1.55-7.39; *P* = 0.002) remained independently associated after multivariable adjustment. ICU admission may represent a composite marker of greater illness severity, frequent invasive procedures, antimicrobial exposure, and factors that may facilitate the transition from previous MDRO isolation to BSIs. This independent association between ICU exposure and subsequent BSI among MDRO carriers is consistent with previous studies [10,17]. The independent association with central venous catheterization is particularly noteworthy, given that intravascular devices provide a potential portal of entry and a surface for microbial adherence. Previous studies have identified central venous catheterization as an important risk factor for BSIs caused by MDRO pathogens [17]. Furthermore, the risk for BSI in patients with colonization appears to increase substantially with cumulative clinical risk factor burden. However, we emphasize that this finding is exploratory and should not be interpreted as a validated prediction score.

Pathogen-specific analysis was restricted to CRKP because it was the only MDRO subgroup with a sufficient number of matched case sets to support a meaningful multivariable analysis. Among the patients with previous CRKP isolation, central venous catheterization was strongly associated with subsequent CRKP BSI in the multivariable model (adjusted OR 7.72, 95% CI 2.44-24.50; *P* < 0.001). This observation is consistent with that of previous reports [17]. Collectively, these findings highlight central venous catheter exposure as a potentially modifiable factor in the progression from previous MDRO isolation to BSI and support rigorous assessment of the ongoing indication for central venous access, adherence to catheter insertion and maintenance bundles, and prompt removal of unnecessary central venous catheters as potentially important components of infection prevention strategies in patients with MDRO colonization.

This study has several limitations. First, its retrospective case-control design precludes definitive causal inference, may be subject to residual or unmeasured confounding factors, and limited our ability to reliably distinguish colonization from infection for MDROs recovered from clinical specimens. Second, this was a single-center study in which MDRO surveillance screening was restricted to a specific patient population, potentially introducing selection bias and limiting the generalizability of the findings to other patient populations and health care settings. Third, single-site, culture-based surveillance may have limited sensitivity for detecting MDRO carriage.

Fourth, molecular typing was not performed to establish clonal relatedness between colonizing and bloodstream isolates. Finally, the association between antibiotic exposure and severity of illness with progression to BSI was not examined. Despite these limitations, this study provides valuable insights into the factors associated with the progression from MDRO isolation to BSI, making the findings particularly relevant for risk stratification among known MDRO carriers.

## Conclusion

The study demonstrated that progression from MDRO isolation to BSI is a clinically significant event associated with prolonged hospitalization and substantially higher mortality. Recent ICU admission and central venous catheterization were independently associated with progression to BSI. These findings may help identify MDRO carriers at greatest risk of BSI and underscore the importance of optimizing potentially modifiable exposures, particularly, the use of central venous catheters. Prospective studies incorporating antimicrobial exposure, illness severity, and molecular characterization are warranted to validate these findings and refine risk stratification strategies.

## Declarations of competing interest

The authors have no competing interests to declare.

## Funding

This research did not receive any specific grant from funding agencies in the public, commercial, or not-for-profit sectors.

## Ethical approval

This study was approved by the medical research ethics committee of the College of Medicine and Health Sciences, Sultan Qaboos University (Ref. No. SQU-EC/109/2025/MREC No. 3626).

## Declaration of generative AI and AI-assisted technologies in the writing process

During the preparation of this work, the authors used Consensus AI to identify, screen, and extract insights from peer-reviewed literature. After using this tool, the authors reviewed and edited the content as needed and take full responsibility for the content of the published article.

## CRediT authorship contribution statement

**Jinan Al Jadeedi:** Writing – original draft, Investigation, Formal analysis, Data curation. **Majada Allouyahi:** Writing – original draft, Investigation, Formal analysis, Data curation. **Rouya Al Busaidi:** Writing – original draft, Investigation, Formal analysis, Data curation. **Shatha Al Riyami:** Writing – original draft, Investigation, Formal analysis, Data curation. **Abdullah Balkhair:** Writing – review & editing, Writing – original draft, Supervision, Project administration, Methodology, Formal analysis, Conceptualization.
